# A cross-sectional study of contraception and abortion utilization among female sex workers in the democratic Republic of Congo, Kenya, and Nigeria: preferences, access, and barriers

**DOI:** 10.3389/frph.2026.1789135

**Published:** 2026-10-29

**Authors:** Regan A. Moss, Isabella Danel, Grace Kamau, Wendy L. Macias-Konstantopoulos

**Affiliations:** 1 Global Health Promise, Portland, OR, United States; 2 Social, Behavioral, Population Health Sciences, Weatherhead School of Public Health, Tulane University, New Orleans, LA, United States; 3 Adjunct Assistant Professor, Community Health Sciences, School of Public Health, University of Illinois, Chicago, IL, United States; 4 African Sex Workers Alliance; 5 Center for Social Justice and Health Equity, Department of Emergency Medicine, Massachusetts General Hospital, Harvard Medical School, Boston, MA, United States

**Keywords:** abortion, contraceptive access, female sex workers, reproductive health, sex work

## Abstract

Female sex workers (FSW) report high rates of unintended pregnancy and unmet need for contraception. Using convenience sampling, this cross-sectional study included 853 FSW recruited from eight cities in the Democratic Republic of the Congo (DRC), Kenya, and Nigeria, where proportionate mortality from unsafe abortion is high. The study examined contraceptive prevalence, preferences, access, and barriers, and abortion history, methods and complications. Non-condom modern contraceptive prevalence was higher among FSW in Kenya (73.2%) than in the DRC (40.8%) and Nigeria (26.9%). A greater proportion of FSW in Kenya reported no abortion in the past five years (82.8%) compared with the DRC (54.0%) and Nigeria (33.9%). Among those reporting abortion, the mean number was lower in Kenya (1.5) than in the DRC (1.7) and Nigeria (2.2). Cost was a common barrier to accessing contraceptives in the DRC and Nigeria (approximately one-third of respondents) but less common in Kenya (9%). Contraceptive preferences differed. The majority of FSW in Kenya preferred long-acting reversible contraception (LARC). Although the majority of FSW in the DRC and Nigeria preferred condoms, client dynamics in criminalized settings may limit consistent condom use making them ineffective. Improving access to free or affordable non-condom modern contraceptives, particularly LARC, is critical to reducing unintended pregnancies and preventing morbidity and mortality from unsafe abortion among FSW. Increased education and counseling on LARC methods may further support uptake.

## Introduction

Family planning, including access to abortion and contraceptives, is recognized in the Sustainable Development Goals (SDG) as necessary to end poverty (SDG 1) and achieve gender equality (SDG 5) (1). Agency over reproductive decision-making, including pregnancy timing, intention and outcomes, is fundamental to reproductive and mental health. As a result of the lack of legal protections surrounding their work and barriers to effective contraceptives, female sex workers (FSW) are particularly vulnerable to unintended pregnancy. If pregnancy occurs, safe abortion options are also limited (2). While high rates of unintended pregnancy among FSW have been described (3), relatively little research has focused on their contraceptive preferences or preferred methods for terminating pregnancy.

Unintended pregnancy among FSW has been linked to sexual exploitation (34) and economic precarity (35) including the costs of child-rearing, potential abandonment by “emotional partners' (35), and loss of clients and income (4). Centering the reproductive health needs and perspectives of FSW is therefore essential for developing patient-centered and responsive family planning services.

### Contraceptive use

Female sex workers report similar desires for parenthood as non-sex working women, yet they often have limited control over pregnancy timing due to structural and interpersonal factors, including client-perpetrated reproductive coercion and the criminalization of sex work (4–6). The structural and social conditions surrounding sex work may elevate risk for unintended pregnancy (3, 7, 8).

Male condom use, the most commonly used form of pregnancy prevention among sex workers is frequently inconsistent: less than 5% of FSW report consistent condom use with clients (9) and approximately one in four FSW in sub-Saharan Africa report unmet contraceptive needs (10). Uptake of long-acting reversible contraception (LARC) is generally low, with reported median use of 1.4% for intrauterine devices (IUD), 6.2% for implants, and 10.3% for injectable contraceptives (11). While programs for FSW may focus on increasing condom use for the prevention of sexually transmitted infections (STIs) including HIV, they neglect the broad range of family planning needs of FSW (12).

Structural and social factors further constrain contraceptive use. The criminalization of sex work increases vulnerability to coercion and limits legal recourse when clients refuse condom use (6). In some contexts, possession of condoms may be used as evidence of “criminality’ and discourages their use (13–15). Financial pressures may also lead FSW to accept higher paying condomless sex, particularly among women supporting children (4).

In addition, condom use is often incorrect and/or inconsistent at comparable rates with both paying and non-paying partners (16–20). Dual protection—using condoms alongside another effective contraceptive method—is recommended to prevent both pregnancy and HIV/STIs (21). Yet fewer than 9% of FSW report using dual methods thus rendering condom use the predictor of unplanned pregnancy for many FSW (10).

FSW report discrimination in clinical care settings and cost as barriers to accessing LARCs (4). Criminalization of sex work and sex work stigma also create barriers.

to contraception access within clinical settings (2). Low health literacy, lack of access to healthcare services and lack of familiarity with modern contraceptive methods further reduce uptake (12, 22). Even when awareness exists, structural barriers such as clinic wait times, fears of HIV testing, and concerns about losing clients may limit use (12).

Despite extensive research on barriers to contraception, relatively little is known about contraceptive preferences among FSW. While condoms are widely promoted for HIV prevention and may be more accessible for FSW than other forms of contraception, they provide limited pregnancy protection when used inconsistently and their use is often outside women's control (6, 13–15). Methods that do not require partner participation, such as LARCs, may offer more reliable pregnancy prevention for FSW in restrictive contexts, due to client power dynamics and criminalization of sex work (4, 6).

### Abortion use

Limited reproductive autonomy and high rates of unintended pregnancy contribute to elevated abortion rates among FSW in many settings (11, 23). In Ethiopia, for example, approximately half of FSW report having had at least one abortion (36), and estimates suggest that 16%–85% of pregnancies among FSW in low- and middle-income countries end in abortion (3). However, relatively little research has examined the methods used.

Legal restrictions on abortion disproportionately affect marginalized populations, including FSW. A global meta-analysis found that where abortion is illegal, 42.8% of FSW report self-managing an abortion (11). While self-managed abortion can be safe with appropriate medication and information, lack of access to regulated medication and accurate

health information may increase risks. The criminalization and stigmatization of sex work further limit access to safe abortion and post-abortion care (2). The legality of abortion is a strong predictor of abortion mortality as people in need of abortions in countries where it is illegal often resort to unsafe methods (11, 24).

Barriers to timely abortion care may also increase risks. FSW report delayed recognition of pregnancy, limited social support, and restrictions on movement due to intimate partner violence (25, 26). Substance use and other socioeconomic factors may further delay care (25). When abortions occur later in pregnancy or involve unsafe methods, the risk of severe complication and death increases. Evidence from the Democratic Republic of the Congo (DRC), Kenya, and Nigeria underscores this risk. A 2019 study of causes of death among FSW in these countries found that unsafe abortion accounted for 57% of maternal deaths and 35% of all deaths among FSW (27). Reported methods among participation from the DRC, Kenya, and Nigeria included ingestion of unknown herbs or medications and invasive “traditional” practices (28).

Recognizing that barriers to effective contraception for FSW result in unplanned pregnancies that often lead to unsafe abortions and preventable maternal deaths, in this study we examine contraceptive use, preferences and barriers among FSW in the DRC, Kenya, and Nigeria. We also examine abortion utilization including methods used and sources of access. By characterizing reproductive health practices and constraints in these settings, this research aims to inform interventions that better address the reproductive health needs of FSW and reduce preventable maternal deaths.

## Methods

This study is part of a broader investigation into the reproductive and maternal health outcomes and experiences of FSW and the health of their children in the DRC, Kenya, and Nigeria. The study design was cross-sectional using a convenience sample. The present study focused on answering four research questions currently unaddressed in the literature: (1a) *What form of contraception do FSW use in the DRC, Kenya, and Nigeria?* (1b) *What barriers exist to accessing their preferred method?* (2a) *What abortion methods do FSW utilize in the DRC, Kenya, and Nigeria?* (2b) *What complications arise from these methods?*

### Study setting and participants

The study design included three cities per country, enrolling 100 FSW per city. Cities were selected based on the recommendations and expertise of local sex worker organizations and nongovernmental organizations (NGO) and prior successful collaboration conducting research with FSW populations in these locations (27) Selection considered the presence of sizable FSW populations, logistical feasibility, and safety for study personnel. Data were collected between February 2022 and February 2023 in eight cities across the DRC (Bukavu and Kinshasa), Kenya (Kisumu, Mombasa and Nairobi), and Nigeria (Abuja, Calabar and Lagos).

Due to social unrest, data collection in Goma (DRC) was halted, and the Kinshasa sample was increased to 150 to help compensate.

Individuals were eligible to participate if they were 18 years of age or older; had engaged in sex work full-time for at least three years; and had one child aged five years of age or younger (to ensure adequate data for the maternal and child health components of the study).

### Recruitment

Participants were recruited by staff from local sex worker-led organizations providing resources to FSW in Kenya and Nigeria and local non-government organizations (NGOs) providing services to FSW in the DRC. As these organizations regularly did outreach in brothels or hot spots, they were able to identify local spots to recruit FSW. The staff from the local

partner organizations would go to a different hotspot within the city every night and share information about the study with FSW. They would screen potential participants at the hotspot and invite eligible FSW to the interview venue (i.e., the NGO office) the next day where they would receive additional information about the study and were consented if they chose to participate. Local partners ensured no FSW were enrolled twice, and they went to a different location each night to recruit new participants.

### Data instruments

Data were collected through an interviewer-administered survey which was based on validated questions from the Demographic and Health Surveys (DHS) (29). The survey collected data on participants' demographics, current use of contraception, and for those who had at least one abortion within the past five years, information about the most recent abortion. The survey was reviewed by local partners (i.e., staff members of sex-worker led organizations), refined based on their feedback, and translations were provided in the DRC.

### Variables

The study instrument included three questions on contraception: (1) *What method of birth control do you use now to prevent pregnancies?* (2) *What method is your preferred method of birth control to prevent pregnancies?* (3) *What problems do you have getting your preferred form of birth control?*

Participants were also asked about abortion use, method, and location: (1) *In the past 5 years, how many abortions have you had?* (2) *For your most recent abortion, what method did you use?* (3) *If you used a pill [during the most recent abortion], what was the name of the pill?* (4) *[During the most recent abortion] Where was the abortion performed (for surgical abortions only)?* As existing studies report that many FSW do not access safe abortions we also asked about complications: (5) *Within the 3 days following your [*most recent] *abortion, did you experience any of the following: fever, excessive bleeding, or infection?* The data instrument presented the question on complications following their most recent abortion as three separate questions.

### Data collection

Non-governmental organizations and sex worker-led organizational staff were trained to administer the study instruments by the lead researcher. Prior to participation, information about the study was presented to potential participants. Participants consented by providing an “X” or check mark on the consent form. To ensure privacy, no identifying information on participants was collected.

Participants completed a structured 60minute interviewer-administered survey. The survey was administered by local staff members of sex-worker led organizations to help facilitate interviewer-participant trust. Interviews were conducted in English in Kenya and Nigeria, and Swahili in Bukavu, DRC and Lingala in Kinshasa, DRC. During the interview, participants received a meal, free childcare, and compensation based on the recommendation of the local partner (approximately $20). Completed questionnaires were reviewed for missing responses and legibility before the participant left the venue. Questionnaires from Kenya and Nigeria were entered into Qualtrics and then uploaded into Excel; in the DRC, data were entered directly into Excel. Differences were due to the capacity of local staff. Following data entry, data were reviewed for completeness and accuracy and cleaned by the lead researcher and research staff.

### Data analysis

Analyses were primarily descriptive and focused on characterizing contraceptive use, barriers, and abortion experiences across the three study countries. Analyses were conducted using IBM SPSS (Statistical Package for Social Sciences) and Microsoft Excel to examine current and preferred contraceptive methods, barriers to accessing preferred methods, and abortion history during the previous five years. Regression analyses were conducted in Stata (StataCorp LLC; version StataNow/BE 19.5 for Mac) to examine within-country associations between current use of non-condom modern contraceptive methods (yes/no) and abortion experience (one or more abortions during the previous 5 years versus none).

For regression analyses, contraceptive methods were classified as non-condom modern (oral contraceptive pills; (e.g., implants, injections, IUDs and female sterilization) or other (condoms, other methods and no method). Although condoms are generally classified as modern contraceptive methods, they were analyzed separately in this study because their effectiveness depends on partner cooperation, an important barrier to their consistent use among FSW.Although the data have a hierarchical structure (individuals nested within cities and countries), multilevel modeling was not undertaken because the number of higher-level units was small (three countries and two to three cities per country). With so few clusters, estimates of random effects and intraclass correlation coefficients would not be reliable.

### Ethics

The study protocol was approved by the Institutional Review Board of Oregon Health & Sciences University (IRB ID: STUDY0002296) and the Ethics Review Committees in each of the three countries, Kenyata University Centre for Research Ethics and Safety in Kenya, Ecole De Sante Public, University de Kenshasha, DRC and Nigerian Institute of Medical Research, Institutional Review Board, Nigeria. All participants gave their written informed consent, which was formally recorded and witnessed by the lead researcher. Our ethical guidelines, standards, and disclosures meet or exceed those required under the Declaration of Helsinki.

## Results

### Participant characteristics

There were 250 participants in the DRC, 302 in Kenya and 301 in Nigeria (Table 1). The target recruitment for each city was 100 FSW. Approximately 100 FSW participated in each of the eight cities, except Kinshasha which had 150. The mean age among participants from the DRC was 24.0; 30.2 in Kenya; and 28.3 in Nigeria. The majority of participants in every country were single/never married. The largest portion of participants in DRC with some education had received secondary education. This was the same in Kenya and Nigeria.

**Table 1:** Participants in the DRC, Kenya, and Nigeria.

Characteristics	DRC Jan-Feb 2023 *n* (%) (*N* = 250)	Kenya Feb-Mar 2022 n (%) (*N* = 302)	Nigeria Mar-Apr 2022 n (%) (*N* = 301)
City Abuja Bukavu Calabar Kinshasa Kisumu Lagos Mombasa Nairobi	- 100 (40.0) - 150 (60.0) - - - -	- - - - 100 (33.1) - 99 (32.8) 103 (34.1)	100 (33.2) - 101 (33.6) - - 100 (33.2) - -
Age			
18–24	140 (56.0)	50 (16.6)	90 (29.9)
25–29	85 (34.0)	97 (32.1	94 (31.2)
30–39	25 (10.0)	136 (45.0)	106 (35.2)
40+	0	19 (6.3)	11 (3.7)
Mean age ± SD	24.0 ± 3.9	30.2 ± 5.8	28.3 ± 6.1
Marital Status Single/never married Married Divorced/Separated Widowed No response	245 (98.0) 0 5 (2.0) 0 0	217 (71.9) 2 (0.7) 64 (21.2) 14 (4.6) 5 (1.7)	221 (73.4) 0 44 (14.6) 34 (11.3) 2 (0.7)
Education level No education Primary Secondary Advanced degree No response	52 (20.8) 80 (31.7) 95 (38.0) 23 (9.2) 0	2 (0.7) 126 (41.7) 142 (47.0) 32 (10.6) 0	17 (5.6) 97 (32.2) 177 (58.8) 9 (3.0) 1 (0.3)

### Contraceptive use

When considering all types of contraceptives (i.e., inclusive of condom use), 6% of participants in the DRC did not use any form of contraception (Table 2). Excluding condoms (male or female), non-use increased to 12.4%. Similarly, 5.9% of respondents in Kenya do not use any contraceptives and non-use increases to 7.2% after excluding condoms. Only 3 of 201 (1.5%) respondents in Nigeria reported that they did not use any contraceptives although this increased to 3.5% when excluding condoms.

**Table 2:** Current birth control method.

Country (*N*)	Non-condome modern contraception									
	Birth control pills n (%)	Female sterilization n (%)	Long-acting reversible contraception			Total non-condom modern contraception n (%)	Condoms n (%)	Other n (%)	None n (%)	NR n (%)
			Implant *n* (%)	Injection *n* (%)	IUD *n* (%)					
DRC (250)	37 (14.8)	20 (8.0)	1 (0.4)	41 (16.4)	3 (1.2)	102 (40.8)	129 (51.6)	4 (1.6)	15 (6.0)	0
Kenya (302)	21 (7.0)	4 (1.3)	61 (20.2)	107 (35.4)	28 (9.3)	221 (73.2)	53 (17.6)	8 (2.6)	18 (6.0)	2 (0.7)
Nigeria (301)	42 (14.0)	2 (0.7)	0	20 (6.6)	17 (5.7)	81 (26.9)	216 (71.8)	1 (0.3)	3 (1.0)	0

“Injectable” includes both single and plural references to birth control injections. “Condom” includes male, female, and joint condom use categories. “Other” includes non-contraceptive methods: abortion, Plan B or emergency contraceptives, the calendar method and other unspecified methods. NR is no response.

In Kenya, the most common current method of birth control was injections (35.4%) followed by implant (20.2%) and condoms (male or female) (17.6%). Female sterilization was not common in Kenya (1.3%). Non-condom modern contraceptive prevalence was 73.2%. In the DRC, 51.6% reported condoms as their primary method of birth control. The next most common method was injection (16.4%) followed by birth control pills (14.8%), and 8% of respondents reported that they had been sterilized. Non-condom modern contraceptive prevalence was 40.8%. The most common method of contraception in Nigeria was condoms (71.8%) followed by birth control pills (17.9%). Non-condom modern contraceptive prevalence was 26.9%.

### Contraceptive preferences

The most preferred contraception method in the DRC (58.0%) and Nigeria (83.1%) were condoms. Excluding condoms, the next most preferred methods were pills and injections in DRC

(12.8% each) and injections in Nigeria (5.7%) followed closely by pills (5.3%). In Kenya, the most preferred method was injections (37.1%) followed by implant (23.5%). Very few participants within any country reported that they had “no preference.” See Figure 1.

**Figure 1:**
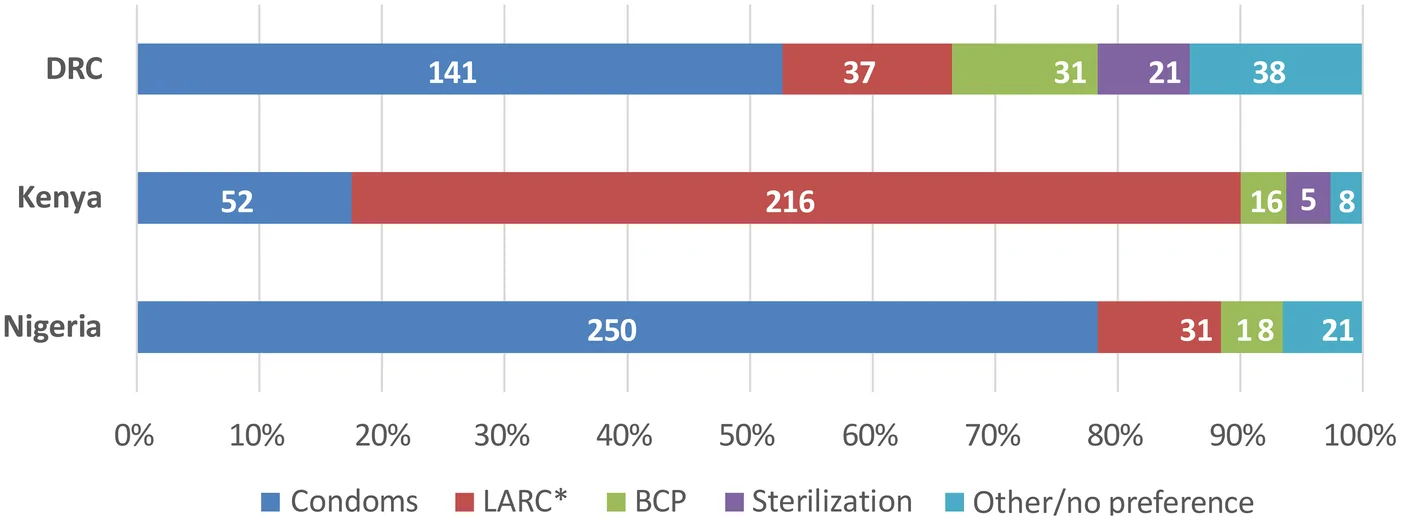
Preferred birth control method. *LARC—long-acting reversible contraception.

### Barriers to accessing preferred methods

A majority of all participants in Kenya (65.65%) and Nigeria (71.4%) reported no issues with accessing their preferred contraceptive, however, over 1 in 3 in Nigeria still experienced barriers (Table 3). In the DRC, the majority (54%) reported experiencing barriers while 46.0% reported that they did not have barriers to accessing their preferred method of contraception. Cost was the most commonly reported barrier to contraceptive access, indicated by 32.4% of participants in the DRC, 23.9% in Nigeria, and 10.9% in Kenya.

**Table 3:** Barriers to accessing preferred birth control methods.

Country	No problems	Cost	Not available	Don't know where to get it	Side effects	Male refusal	Facility hours/ distance	Lack of knowledge	Other	NR/DTA
	%									
DRC (*N* = 250)	46.0	32.4	4.8	3.6	1.2	9.6	0	0.2	0.8	0.4
Kenya (*N* = 302)	65.6	10.9	9.3	0	3.0	0	2.0	4.0	1.7	6.6
Nigeria (*N* + 301)	71.4	23.9	3.0	0.3	0	0	0.3	1.3	0.7	0.3

Categories are grouped for simplicity. “Side Effects” includes reported and feared side effects. The “Cost” combines all economic access barriers. “No Problems” includes responses indicating no issues accessing family planning.

### Abortion experience

Nearly half of respondents (115; 46.0%) in the DRC reported they had had an abortion in the last five years (Table 4). Among them, the mean number of abortions was 1.7 (*M*). In Kenya, 250 (82.8%) respondents had not had an abortion within the last five years, while 47 (15.6%) reported having at least one abortion within the past five years. Among the 47 women who reported an abortion in Kenya, the average number of abortions was 1.5. Among participants in Nigeria, two-thirds of women (65.4%) reported at least one abortion in the last 5 years, and the average number of abortions, among those reporting at least one abortion was 2.2. Only one-third of Nigerian FSW (102; 33.9%) reported they had not had an abortion within the past 5 years.

**Table 4:** Number of abortions in past 5 years.

Country	0	1	2	3	4–5	6–7	>7	NR/NS^a^	One or more abortions in past five years	Total abortions (mean)^b^
	n (%)									
DRC	135	53	36	13	8	5	0	0	115	230
(*N* = 250)	(54.0)	(21.2)	(14.4)	(5.2)	(3.2)	(2.0)			(46.0)	(1.7)
Kenya	250	37	6	3	0	0	1	5	47	68
(*N* = 302)	(82.7	(12.2)	(2.0)	(1.0)			(0.3)	(1.7)	(15.6)	(1.5)
Nigeria	102	68	70	36	17	4	2	2	197	439
(*N* = 301)	(33.9)	(22.6)	(23.3)	(12.0)	(5.6)	(1.3)	(0.7)	(0.6)	(65.4)	(2.2)

a NR/NS, not reported/not specified whether had an abortion or not.

b mean number of abortions in the past five years among FSW who had 1 or more abortions.

### Abortion methods overall

The most commonly reported abortion method in the DRC was a pill (60; 52.2%); the second most common method were local herbs (25; 21.7%). The most common abortion method in Kenya was also a pill (30; 30.9%) and the second was surgical (23.1%). The most common method in Nigeria were herbs (71; 35.7%), followed by surgical methods (60; 30.2%) and pills (27.4%). See Figure 2.

**Figure 2:**
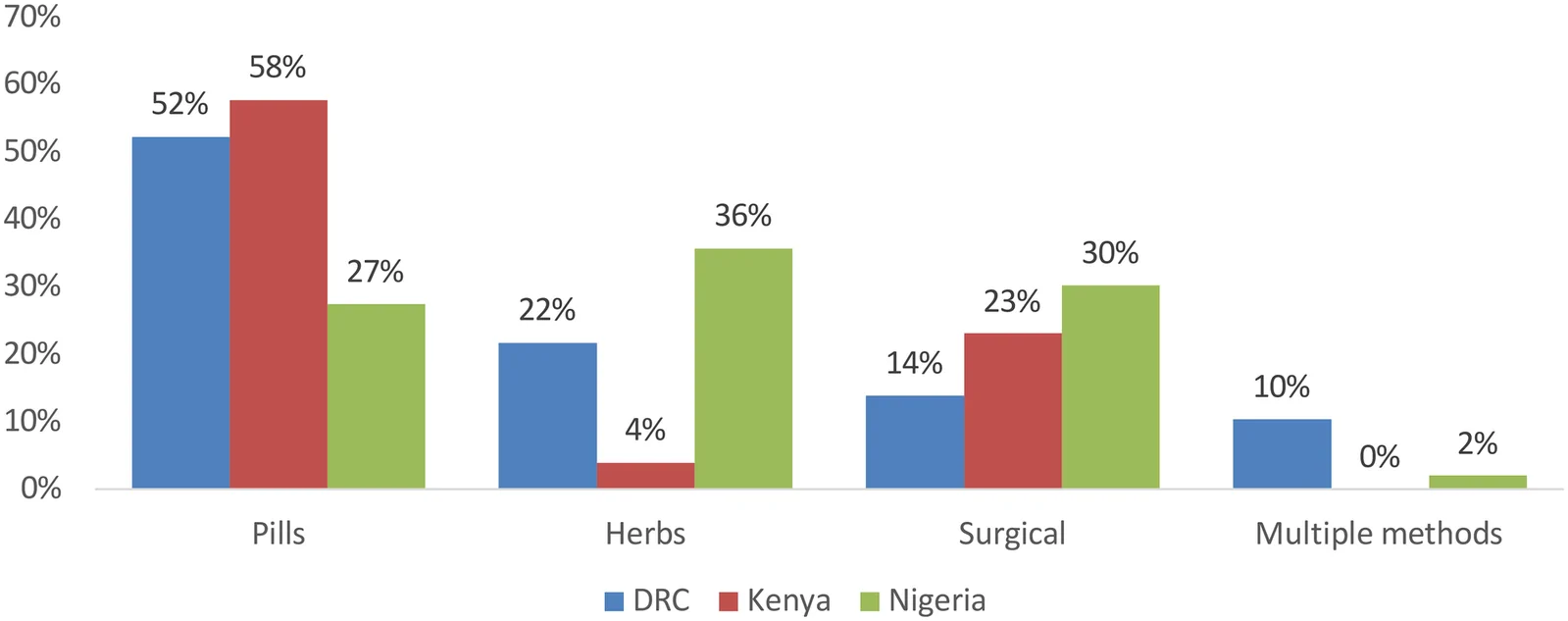
Abortion method for the most recent abortion.

### Abortion pill type

Abortion pills were a common method of abortion in DRC and Kenya and utilized across respondents in all three countries, however, out of 145 respondent who used pills, over a third (53; 36.36%) did not know the name of the pills they consumed. Of 60 participants who used abortion pills in DRC, the majority of respondents (37; 61.7%) reported using misoprostol. In Kenya, 30 respondents (57.7%) reported using abortion pills., However, only 7 stated they used misoprostol and nearly two-thirds (19; 63.3%) did not know the type of pills they consumed. Not all pills were abortifacients and included among these were quinine (malaria treatment), decaris (a parasitic drug), plan B or “after pills”, and other non-controlled, non-abortifacients (e.g., ginger).

### Abortion locations

Of the 51 participants who reported abortion locations in the DRC, 21 (41.2%) had an abortion at home, the most reported location. Of 89 respondents in Nigeria who reported an abortion location, 51 (57.3%) had an abortion in a “quack's” office (i.e., a fake medical doctor). Of the respondents who reported an abortion location in Kenya, the majority were in other locations (18) or in a “quack's” office (17).

### Self-reported abortion complications

The majority of the 361 participants who reported an abortion (272; 75.3%) also reported a fever after their most recent abortion. Of the 272 participants who experienced fevers, 127 (46.7%) reported an abortion location and 66 (52%) of these abortions were administered in a “quack's” office. A total of 269 respondents (74.5%) reported excessive bleeding after their most recent abortion, and of the 119 (44.3%) who also reported an abortion location, 53 (44.5%) of these abortions were administered in a “quack's” office. Finally, of the 193 participants (53.5%) who reported an infection, 97 (50.3%) reported a location and 46 (47.4%) of abortions were administered in a “quack's” office.

### Non-condom modern methods and abortion experiences

In country-specific analyses, there was no statistically significant association between non-condom modern contraceptive methods and reported abortion in the past five years in any of the three countries. Estimated reductions in five-year abortion prevalence associated with use of non-condom modern contraceptive methods were 30 percentage point lower probability of reporting an abortion in DRC (*p* = 0.146), 10 percentage points lower in Nigeria (*p* = 0.405), and 3 percentage points lower in Kenya (*p* = 0.576). Current contraceptive use was measured at the time of the survey, whereas abortion experience was assessed over the preceding five years. Therefore, some participants reporting an abortion may have initiated contraception after their abortion.

## Discussion

This study examined contraceptive prevalence, barriers to accessing preferred methods of contraception, and abortion experience among FSW in three African countries where abortion-related mortality among FSW is high. Our findings indicate substantial differences across countries. FSW in Kenya reported substantially higher use of non-condom modern contraception, such as injections and implants, than those in Nigeria and the DRC, and far fewer reported abortions within the past five years. In Kenya, non-use of contraception was relatively low (6.0%), with injections (35.5%) and implants (20.2%) the most commonly used methods. In contrast, respondents in the DRC and Nigeria primarily used condoms as their main contraceptive method (51.6% and 71.8%, respectively).

### Contraceptive preferences

Very few FSW across the three countries reported having “no preference” for contraception (0.7%–2.9%). Although the survey did not assess reasons for their preferences, reproductive health programming should prioritize the choices of FSW while ensuring they have accurate information about the full range of contraceptive options. Notably, the most commonly preferred contraceptive methods in Kenya were LARCs, particularly injections and implants.

Conversely, condoms were the most commonly preferred method in the DRC and Nigeria—58.0% and 83.0%, respectively. Non-condom modern methods are not only the most effective biologically (30) but for FSW, LARCs offer greater protection against unintended pregnancy because they do not require partner participation and therefore may reduce the impact of financial coercion or client refusal of condom use.

Public health guidance emphasizes dual protection. While it is recommended that FSW use condoms to protect against HIV/STIs (21), effective contraception to prevent unintended pregnancy is also needed. As described, the social and structural conditions surrounding sex work may elevate risk for unintended pregnancy (3, 7). Effective programming for FSW would provide education on the benefits of dual methods: condoms prevent STIs, and effective contraceptives prevent unplanned pregnancies and improve agency in pregnancy timing (31). Prior research in Kenya reported that fewer than 20% of FSW used modern contraceptives (12), whereas the present study suggests substantially higher uptake among Kenyan FSW. Although differences in study populations may contribute to this discrepancy, the findings may also reflect improvements in contraceptive access or health literacy in recent years.

Compared with sexually active unmarried urban women in recent DHS surveys, FSW in all three countries reported higher use of contraception, including higher use of non-condom modern contraceptive methods. This finding suggests that limited contraceptive uptake alone does not explain the high rates of unintended pregnancy and abortion reported among FSW. Rather, the type of method used and the challenges of relying on condoms in the context of sex work may be particularly import considerations.

Participants differed demographically across countries: those in the DRC were, on average, younger, more often single/never married, and more likely to have no formal education than participants in Kenya or Nigeria. These differences should be kept in mind when interpreting the cross-country patterns, as younger age, unmarried status, and lower educational attainment have been linked to reduced contraceptive knowledge and access, and may partially underlie some of the differences observed.

### Contraceptive barriers

Cost emerged as the most prominent barrier to accessing preferred contraceptive methods, particularly in the DRC and Nigeria. Approximately one-fourth to one-third of respondents in these countries reported cost-related barriers. In contrast, only about 10% of respondents in Kenya reported cost-related barriers. This finding is consistent with a previous report indicating that the Government of Kenya provided contraceptive methods free of charge at public health facilities (32). However, access barriers may still exist in other service settings such as private clinics or pharmacies. Reducing financial barriers to contraception, ideally irrespective of the service setting, may therefore represent an important strategy for improving reproductive health outcomes among FSW.

### Abortion experience

Participants in Nigeria reported substantially more abortions in the previous five years than those in the DRC and Kenya, and the mean number of abortions was also higher. Interpretation of the association between non-condom modern contraceptive use and abortion is limited by the temporal structure of the data. The lack of statistically significant associations may reflect temporal misalignment between exposure and outcome. Contraceptive use was measured at the time of the survey, whereas abortions were reported over the preceding five years. As a result, current contraceptive use may not reflect use at the time pregnancies occurred. Some women may have initiated non-condom modern contraceptive methods following an abortion, which would bias associations toward the null. This temporal mismatch may attenuate true associations and contribute to imprecise estimates, making it more difficult to detect statistically significant relationships even when underlying differences exist. Within this context, the higher number of abortions observed in Nigeria may still be consistent with greater reliance on condoms as a primary method.

As unsafe abortion is one of the leading causes of death among FSW (28), there is a challenge to balance FSW preference for condoms, which are ineffective given the high rates of unintended pregnancies, and unsafe abortions. Thus, NGOs that provide sexual and reproductive health should help FSW to use condoms more effectively, so that programming remains culturally congruent, as well as educate FSW about alternative, more effective, contraceptive options so they have the reproductive health information needed to make informed decisions. Where low health literacy is driving contraceptive preferences that are less effective given their circumstances, outreach can address misconceptions and barriers to LARCs and identify the challenges and limitations that FSW experience around non-condom modern contraceptive methods and use of dual methods. Improving health literacy may lead to increased desire for and uptake of dual methods.

In addition, there is a dire need to address the criminalization of sex work as well as the ubiquity of unsafe abortion methods offered to women. Increasing FSW's access to free or affordable effective methods of contraception is the most preventative and life-saving strategy. While a shocking number of FSW deaths from unsafe abortion has been documented (28), we also note that providing them with access to free or affordable effective methods of contraception may reduce unintended pregnancy rates, prevent the significant burden of morbidity and mortality from unsafe abortions, and reduce the number of children by their mothers' death from an unsafe abortion. As cost is the leading barrier to contraceptive methods among FSW, policies and programs aimed at reducing mortality among FSW, of which unsafe abortion is a leading cause, should prioritize cost related barriers.

The majority of participants in the DRC and Kenya used a pill to induce an abortion but many did not know the active ingredients, if any, of the pill. While medications such as mifepristone and misoprostol are safe and effective for medication abortion when used appropriately, limited access to regulated pharmaceutical products and reliable information may result in the use of ineffective or unsafe substances, or inappropriate and potentially dangerous use late in gestation. Many respondents likely did not have access to regulated abortifacients and those who did may have used them late in gestation.

Abortion laws in the three countries studied are restrictive, with only narrow legal exceptions that do not include unintended pregnancy (33). In such settings, individuals seeking abortions often rely on informal or unregulated providers. In this study, many participants reported obtaining abortion services from individuals described as “quack” providers—individuals presenting themselves as medical professionals without appropriate training or credentials. These circumstances likely contribute to the high rates of complications reported by participants.

Structural factors—including legal restrictions on abortion, limited access to regulated medication, and barriers to clinical care—may therefore increase the likelihood that FSW resort to unsafe abortion methods. Improving access to affordable contraception may help reduce unintended pregnancy and the need for abortion, particularly in settings where safe abortion services remain legally or practically inaccessible.

Future research should further examine factors shaping contraceptive preferences and abortion decision-making among FSW. Additional work is also needed to better understand knowledge and attitudes regarding LARC methods, barriers to dual method use, reasons for seeking abortion, experiences with clinical reproductive health services, and access to post-abortion care.

### Limitations

FSW are a hard-to-reach population, making it difficult to construct a sampling frame.

This study relied on a convenience sample, and although participants were recruited from multiple cities and venues, the findings are not generalizable to all FSW in the DRC, Kenya, or Nigeria. The participants included in this study may also differ in important ways from other FSW populations, including those working in rural areas or in different types of work environments.

The eligibility criteria required participants to have at least one child under the age of five, as the broader study examined maternal and child health outcomes. As a result, FSW without young children were excluded. This criterion may have introduced selection bias if women without young children have greater use of effective contraception or greater use of abortions.

There are important temporal limitations in the study design. Current contraceptive use was measured at the time of the survey, whereas abortion experience was assessed over the previous five years. Because non-condom modern contraceptive use may change over time—particularly following an unintended pregnancy or abortion—the exposure and outcome are not temporally aligned. This limits the ability to determine the direction of the association and may bias estimates toward the null, contributing to imprecision and lack of statistical significance. These findings should therefore be interpreted cautiously. Longitudinal studies measuring contraceptive use and pregnancy outcomes over the same time period are needed to better understand these relationships.

Additionally, the survey did not assess the reasons underlying contraceptive preferences or non-preference, which limits interpretation of the findings. Finally, the study did not collect information on how the COVID-19 pandemic may have influenced contraceptive access or abortion care. Because the date of the most recent abortion was not recorded, we were unable to assess potential changes in abortion experiences before and during the pandemic.

## Conclusion

Supporting family planning is essential for advancing the reproductive rights and health of FSW. Agency over reproductive decision-making—including pregnancy timing, intention, and outcomes—is fundamental to the wellbeing of FSW and their families. This study highlights substantial differences in contraceptive use and abortion experiences among FSW across the DRC, Kenya, and Nigeria. The findings underscore the importance of expanding access to affordable non-condom modern contraceptive methods and strengthening education about effective contraceptive options. Increasing access to contraception may help reduce unintended pregnancies and decrease reliance on unsafe abortion methods.

Given the legal restrictions surrounding abortion in many settings, improving access to effective contraception represents one of the most feasible strategies for reducing abortion-related morbidity and mortality among FSW. However, FSW will continue to need abortion care, and access to safe procedures as well as abortion education is critical to supporting their full range of family planning needs. Policies and programs that ensure free or affordable contraception, alongside reproductive health education and respectful care, are critical to protecting the health and lives of FSW and preventing avoidable deaths from unsafe abortion.

## Data Availability

The datasets presented in this article are not readily available because data access can be discussed through Global Health Promise. Requests to access the datasets should be directed to bwillis@globalhealthpromise.org, Brian Willis.
